# Streptococcus‐related acute suppurative thyroiditis in a COVID‐19‐positive child: A rare case report

**DOI:** 10.1002/ccr3.6812

**Published:** 2023-01-03

**Authors:** Elham Maleki, Kimia Iranmanesh, Mohammad Javad Najafzadeh, Amir Baniasad

**Affiliations:** ^1^ Endocrinology and Metabolism Research Center Institute of Basic and Clinical Physiology Science, Kerman University of Medical Sciences Kerman Iran; ^2^ Student Research Committee Kerman University of Medical Sciences Kerman Iran

**Keywords:** acute suppurative thyroiditis, COVID‐19, nonhemolytic streptococcus

## Abstract

In this case report, we present a 10‐year‐old girl with acute suppurative thyroiditis (AST) symptoms, such as fever, sore throat, and swelling in the suprasternal region, who had a positive PCR test for COVID‐19. The result of the secretions culture obtained from the abscess drainage was positive for nonhemolytic Streptococcus.

## Graphical abstract

The association between acute suppurative thyroiditis (AST) and COVID‐19 is rare but very important. The early treatment and diagnosis of AST, with complete aspiration and antibiotic therapy, can induce full recovery in patients without surgery.

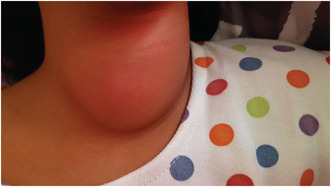

## 1 INTRODUCTION

Bacterial co‐infections and superinfections are common in respiratory viral infections such as COVID‐19.^1^ Viral infections can weaken host immunity and facilitate the development of viral–bacterial infections.^2^ Following COVID‐19‐induced immunosuppression, most hospitalized patients with COVID‐19 developed a secondary bacterial infection during hospitalization.^3^

Acute suppurative thyroiditis (AST) is a rare and potentially life‐threatening endocrine emergency. The incidence of AST is 0.1%–0.7% of all thyroid diseases.^4^ The presence of iodine and hydrogen peroxide in the structure of the thyroid gland as well as its anatomical features, such as capsular lining and lymphatic drainage, are the mechanisms proposed to inhibit and prevent AST.^4^

So far, several cases of AST by the rare pathogens have been reported.^5^ The outcomes of AST have varied from complete recovery and euthyroidism to a mortality rate of 7.9% or more in the absence of immediate intervention.^5^ Consequently, early diagnosis and treatment of suspected cases of AST are essential to reduce mortality from the disease. In addition, AST patients with poor outcomes were less likely to be reported due to bias toward writing negative consequences. Few articles offer an empirical approach to defining ideal diagnostic and management strategies for the optimal patient outcome.^5^, ^6^

At the end of 2019, a new type of COVID‐19 with the primary manifestation of pneumonia was identified in China and quickly caused a worldwide pandemic.^7^ Extrapulmonary manifestations of the COVID‐19 include acute kidney injury, thrombosis in various organs, hepatocellular injury, and neurological and dermatological disorders.^8^ Severe acute respiratory syndrome coronavirus 2 (SARS‐CoV‐2) enters body cells by binding to angiotensin‐converting enzyme 2 (ACE2) receptors and causes COVID‐19.^8^, ^9^ ACE2 receptors are also present in thyroid tissue, and so far, the coexistence of some thyroid diseases, including subacute thyroiditis, with COVID‐19 has been reported.^8^, ^9^

In this study, we investigated the clinical manifestations, course of diagnosis, treatment, and outcome of a case of acute bacterial thyroiditis concurrent with COVID‐19 in a 10‐year‐old patient.

## 2 CASE HISTORY

The patient was a 10‐year‐old girl with a non‐persistent fever 2 weeks before admission, which improved with acetaminophen. The patient gradually developed a sore throat and swelling in the suprasternal region. Despite taking an antibiotic (cephalexin) under a doctor's supervision, there was no improvement; the patient's swelling and pain increased, and neck movements were painful. The patient had redness in the swelling area from the morning of admission and was referred to our third‐level hospital.

The patient's parents had experienced symptoms of COVID‐19, including fever, cough, and myalgia, in the preceding 2 weeks, but the patient had no recent history of sneezing, coughing, or neck trauma. The patient did not have any medical problems or drug history prior to the thyroiditis. The patient's family does not have any past medical history of autoimmune or infectious diseases. The patient was born by cesarean section (term labor). The parents were not related to each other. The growth and development of the patient were favorable. The patient took her vaccines entirely as per the national protocol of vaccination. The patient had no history of COVID‐19 vaccination.

**FIGURE 1:**
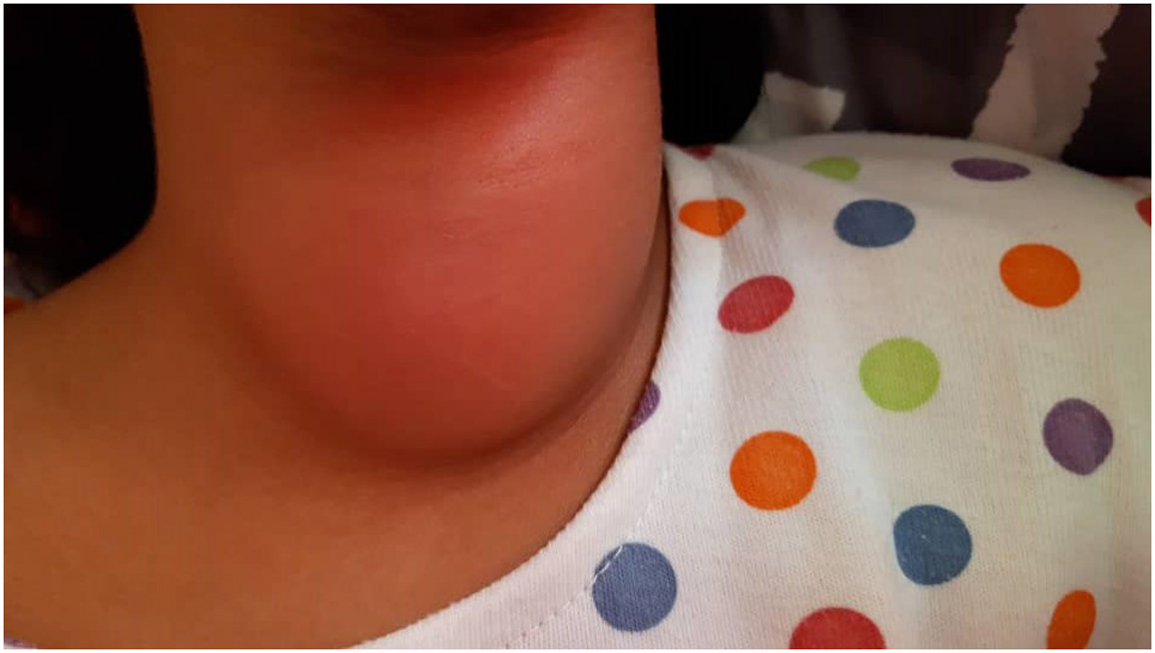
Anterior view of the patient's neck at time of admission. Acute suppurative thyroiditis has caused redness and swelling in the patient's neck.

Upon entering the hospital, the patient had a heart rate of 120, respiratory rate of 17, blood pressure of 90/60 mmHg, temperature of 38.5°C, and oxygen saturation of 97%. Her weight was 26 kg (between the 5th and 10th percentile), and her height was 128 cm (between the 5th and 10th percentile). During examination of the patient's head and neck, a swollen, red, warm, and tender area measuring 5 × 6 cm was found in the front of the neck, in the thyroid gland area, which limited the patient's neck movements due to its pain (Figure 1). Erythema was also observed in the patient's pharynx examination. Despite the fact that the thyroid was significantly painful to the touch, the patient allowed a superficial examination, but did not cooperate for a detailed examination with more pressure.

Physical examination revealed that the patient had submandibular and cervical lymphadenopathy, and nasal inspection revealed hypertrophy of the lower concha bilaterally. The lungs were clear to auscultation. No other pathological findings were found.

In the laboratory tests performed upon arrival, the patient had leukocytosis (white blood cells (WBC) = 18.3 × 10^3^/μl [normal range = 4–10]), increased ESR (65 mm/h [normal range ≤ 22 mm/h]), and positive blood culture (Pseudomonas). The patient had TSH = 0.605 mIU/L (normal range = 0.6–4.48) and T4 = 12.1 μg/dl (normal range = 15–6.4). The patient's PCR test result for COVID‐19 was positive (Table 1).

**TABLE 1:** Laboratory results of the patient at time of admission

Test	Result	Unit	Reference value
WBC	18.3	×10^3^/μl	4–10
Absolute neutrophil count	16.2	×10^3^/μl	‐
Lymphocyte	1.3	×10^3^/μl	‐
RBC	4.5	×10^6^/μl	4.2–5.4
Hb	12.3	gr/dl	14.0–18.0
Hct	36.2	%	39–52
MCV	79.7	fl	80–100
Platelet	756	×10^9^/μl	150–450
PT	16	Sec	13–15
PTT	40	Sec	24–36
INR	1.4	‐	‐
ESR	65	mm/h	Up to 22
BS	99	mg/dl	<140
Urea	19	mg/dl	15–45
Creatinine	0.5	mg/dl	0.6–1.3
Na	136	mEq/L	135–145
K	5	mEq/L	3.8–5
Ca^+^ Ionized	4.4	mEq/L	4.2–5.2
AST	18	units/L	<31
ALT	8	units/L	<31
ALP	360	units/L	180–1200
TSH	0.605	mIU/L	0.6–4.48
T4	12.1	μg/dl	6.4–15
PCR assays for SARS‐COV‐2	Positive	‐	‐

Abbreviations: ALP, alkaline phosphatase; ALT, alanine aminotransferase; AST, aspartate aminotransferase; BS, blood sugar; Ca, calcium; ESR, erythrocyte sedimentation rate; Hb, hemoglobin; Hct, hematocrit; INR, international normalized ratio; K, potassium; MCV, mean corpuscular volume; Na, sodium; PCR, polymerase chain reaction; PT, prothrombin time; PTT, partial thromboplastin time; RBC, red blood cells; SARS‐COV‐2, severe acute respiratory syndrome coronavirus 2; T4, thyroxine; TSH, thyroid stimulating hormone; WBC, white blood cells.

In the sonography of the patient's neck, the left lobe of the thyroid was heterogeneous and hypervascular. A hetero‐hypo echoic lesion with unclear borders and no vascularity was observed in the middle of the neck, the maximum depth of which was 42 mm. Moreover, numerous reactive lymph nodes with a maximum short axis diameter (SAD) of 4 mm were seen from the neck's left anterior chain, indicating purulent thyroiditis with abscess formation. Biopsy and aspiration of the lesion were performed under ultrasound guidance. The pathology reported the sample taken from the patient was the abscess wall. The result of the culture of the secretions obtained from the abscess drainage was positive for nonhemolytic Streptococcus. However, a PCR test for secretions obtained from the abscess drainage was negative for COVID‐19.

Because of the positive PCR for COVID‐19 results and the patient's clinical condition, the patient was given supportive treatment for COVID‐19. In addition, acute bacterial thyroiditis and purulent abscess were treated by abscess drainage and prescribing vancomycin (300 mg every 8 h) and metronidazole (250 mg every 6 h). Acetaminophen tablets (325 mg) and ibuprofen tablets (200 mg) were prescribed in case of pain.

After 11 days of receiving the above‐mentioned treatment, the patient's symptoms improved, and the repeat of the patient's blood culture was negative. The patient was discharged with a prescription for clindamycin at a dose of 300 mg every 8 h. Two weeks later, the patient went to the clinic for follow‐up, and her clinical condition was completely improved, and symptoms resolved.

## 3 DISCUSSION

Herein, we reported the case of a 10‐year‐old girl with acute purulent thyroiditis caused by simultaneous contraction of nonhemolytic Streptococcus and COVID‐19.

Acute suppurative thyroiditis is a rare but potentially fatal disease, and early diagnosis and treatment are crucial. As with our patient, the most common manifestation is pain and swelling in the neck area.^5^ Other clinical manifestations of this disease include fever, dysphagia, skin erythema, odynophagia, and dysphonia.^5^

Acute suppurative thyroiditis is usually seen in patients with immunodeficiency, thyroid pathology, or anatomic thyroid abnormalities, and reports on its idiopathic type are limited. Cawich et al.^10^ reported a 60‐year‐old man with idiopathic thyroid abscess with no underlying cause; the result of the culture of the secretions obtained from the abscess drainage was positive for Staphylococcus aureus. The most common pathogens causing this disease are aerobic Gram‐positive bacteria, and at the top of the list are Streptococcus spp.^5^ In our study, the result of the culture of aspirations from the abscess was positive for nonhemolytic Streptococcus.

Group B Streptococcus are Gram‐positive bacteria and the leading cause of neonatal sepsis and meningitis in developed countries.^11^, ^12^ Nonhemolytic strains cause severe infections to a lesser extent. In Six et al.'s study on 1776 strains of Group B Streptococcus isolated in France between 2006 and 2013, only 63 were nonhemolytic strains, 47 of which caused severe infections such as bacteremia and meningitis.^12^ Our patient's nonhemolytic Streptococcus had caused AST (an invasive disease).

In late 2019, a type of coronavirus called COVID‐19 caused an epidemic in Wuhan, China. The disease spread rapidly in a large number of countries.^7^ In recently conducted meta‐analyses, bacterial infection in hospitalized patients with COVID‐19 disease was reported as 7%.^13^, ^14^ It is also known that patients with COVID‐19 and bacterial infection have a higher risk of mortality and morbidity.^15^ According to a recent study on 1‐month to 12‐year‐old children with COVID‐19, the bacterial co‐infection rate was 7.7%.^16^

Studies have shown the simultaneous occurrence of some thyroid disorders in COVID‐19 patients.^8^, ^17^ Lania et al.^18^ studied 287 hospitalized patients with COVID‐19 and found that 20.2% of patients had thyrotoxicosis (TSH < 0.33). Previous studies have also reported the association of subacute thyroiditis with COVID‐19,^8^, ^17^ but to the best of our knowledge, the association of acute bacterial thyroiditis with COVID‐19 to date has not been reported. Our patient had COVID‐19 at the same time as AST. So far, several mechanisms have been proposed regarding the association of thyroid diseases with COVID‐19. Severe acute respiratory syndrome coronavirus 2 (SARS‐CoV‐2) causes COVID‐19 in patients through ACE 2 host cell receptors, which are also present in thyroid tissue. The immune response to COVID‐19 in these patients can cause cytokine storms, damage thyroid tissue, and cause susceptibility to thyroid diseases.^8^ Wei et al.^19^ reported damage to thyroid follicular cells and their apoptosis in severe acute respiratory syndrome (SARS) patients, which can justify the higher prevalence of thyroid disorders in COVID‐19 patients. Overall, our patient had normal TSH, and evaluating whether the association of COVID‐19 with AST in our patient was coincidental or not requires further studies.

The base of AST treatment in the past was surgery, but complete aspiration and intensive antibiotic therapy have gradually replaced surgery as the first line of treatment.^5^ Our patient was treated with aspiration and antibiotic therapy and responded to the treatment.

## 4 CONCLUSION

The case reported herein is the first reported case of the co‐occurrence of AST and COVID‐19. Moreover, the COVID‐19 PCR test was negative for abscess drainage secretions despite the positive COVID‐19 PCR test for blood sample. Determining the relationship between these two diseases requires detailed studies if similar cases are reported. Especially in AST cases where the diagnosis and treatment of the illness are made at the right time, complete aspiration and antibiotic therapy can result in patients' full recovery without surgery.

## AUTHOR CONTRIBUTIONS

**Elham Maleki:** Conceptualization; supervision; writing – original draft. **Kimia Iranmanesh:** Writing – original draft; writing – review and editing. **Mohammad Javad Najafzadeh:** Data curation; software; writing – review and editing. **Amir Baniasad:** Investigation; methodology; methodology; writing – original draft; writing – review and editing.

## FUNDING INFORMATION

There is no external funding source for this case report.

## CONFLICT OF INTEREST

None.

## ETHICAL APPROVAL

The therapy procedure has been approved by the “Iran National Committee for Ethics in Biomedical Research” (http://ethics.research.ac.ir/IndexEn.php, no.: IR.KMU.AH.REC.1401.036).

## CONSENT

Written informed consent was obtained from the patient to publish this report in accordance with the journal's patient consent policy.

## Data Availability

The data that support the findings of this study are available from the corresponding author upon reasonable request.
